# Sensing of cytoplasmic chromatin by cGAS activates innate immune response in SARS-CoV-2 infection

**DOI:** 10.1038/s41392-021-00800-3

**Published:** 2021-11-03

**Authors:** Zhuo Zhou, Xinyi Zhang, Xiaobo Lei, Xia Xiao, Tao Jiao, Ruiyi Ma, Xiaojing Dong, Qi Jiang, Wenjing Wang, Yujin Shi, Tian Zheng, Jian Rao, Zichun Xiang, Lili Ren, Tao Deng, Zhengfan Jiang, Zhixun Dou, Wensheng Wei, Jianwei Wang

**Affiliations:** 1 Biomedical Pioneering Innovation Center, Beijing Advanced Innovation Center for Genomics, Peking-Tsinghua Center for Life Sciences, Peking University Genome Editing Research Center, State Key Laboratory of Protein and Plant Gene Research, School of Life Sciences, Peking University, Beijing, China; 2 NHC Key Laboratory of Systems Biology of Pathogens, Institute of Pathogen Biology, Chinese Academy of Medical Sciences and Peking Union Medical College, 100730 Beijing, P.R. China; 3 Key Laboratory of Respiratory Disease Pathogenomics, Chinese Academy of Medical Sciences and Peking Union Medical College, 100730 Beijing, P.R. China; 4 Christophe Merieux Laboratory, Institute of Pathogen Biology, Chinese Academy of Medical Sciences and Peking Union Medical College, 100730 Beijing, P.R. China; 5 CAS Key Laboratory of Pathogenic Microbiology and Immunology, Institute of Microbiology, Chinese Academy of Sciences, Beijing, 100101 China; 6 Key Laboratory of Cell Proliferation and Differentiation of the Ministry of Education, School of Life Sciences, Pseking University, Beijing, 100871 China; 7 Peking-Tsinghua Center for Life Sciences, Peking University, Beijing, 100871 China; 8 Center for Regenerative Medicine, Massachusetts General Hospital, Boston, MA USA; 9 Harvard Stem Cell Institute, Harvard University, Cambridge, MA USA; 10 Department of Medicine, Massachusetts General Hospital, Harvard Medical School, Boston, MA USA

**Keywords:** Innate immunity, Infectious diseases

## Abstract

The global coronavirus disease 2019 (COVID-19) pandemic is caused by severe acute respiratory syndrome coronavirus 2 (SARS-CoV-2), a positive-sense RNA virus. How the host immune system senses and responds to SARS-CoV-2 infection remain largely unresolved. Here, we report that SARS-CoV-2 infection activates the innate immune response through the cytosolic DNA sensing cGAS-STING pathway. SARS-CoV-2 infection induces the cellular level of 2′3′-cGAMP associated with STING activation. cGAS recognizes chromatin DNA shuttled from the nucleus as a result of cell-to-cell fusion upon SARS-CoV-2 infection. We further demonstrate that the expression of spike protein from SARS-CoV-2 and ACE2 from host cells is sufficient to trigger cytoplasmic chromatin upon cell fusion. Furthermore, cytoplasmic chromatin-cGAS-STING pathway, but not MAVS-mediated viral RNA sensing pathway, contributes to interferon and pro-inflammatory gene expression upon cell fusion. Finally, we show that cGAS is required for host antiviral responses against SARS-CoV-2, and a STING-activating compound potently inhibits viral replication. Together, our study reported a previously unappreciated mechanism by which the host innate immune system responds to SARS-CoV-2 infection, mediated by cytoplasmic chromatin from the infected cells. Targeting the cytoplasmic chromatin-cGAS-STING pathway may offer novel therapeutic opportunities in treating COVID-19. In addition, these findings extend our knowledge in host defense against viral infection by showing that host cells’ self-nucleic acids can be employed as a “danger signal” to alarm the immune system.

## Introduction

The outbreak of COVID-19 caused by SARS-CoV-2^1,2^ poses a great threat to global public health. To date, the knowledge on the molecular pathogenesis of SARS-CoV-2 is still limited, thwarting the development of therapeutic and prevention strategies. Since innate immunity plays pivotal roles in both host defense and viral immunopathology, understanding the roles of innate immunity involved in SARS-CoV-2 infection is an important biomedical objective.

Activation of the innate immune system upon viral infection is generally thought to be initiated by recognizing specific viral components, termed as pathogen-associated molecular patterns (PAMPs) that are recognized by host pattern recognition receptors (PRRs).^3^ Viral genetic materials, such as genomic RNA, are detected by RIG-I-like receptors (RLRs), including RIG-I or MDA5, and the Toll-like receptors (TLRs). In contrast, viral genomic DNA exposed in the cytosol is detected by cGAS. Upon activation, RLRs and cGAS stimulate adaptor molecules MAVS and STING, respectively, to activate downstream signaling pathways that eventually trigger the expression of antiviral or immunoregulatory cytokines, such as interferons (IFNs).^4^ In addition to sensing viral components, the RLRs and cGAS pathways are reported to mediate sterile inflammation in the absence of infection induced by self-nucleic acids, such as in autoimmune disorders.^5^ Whether host nucleic acids can mediate antiviral immunity is poorly understood.

SARS-CoV-2, an RNA virus, has evolved multi-level strategies to evade the RLR-sensing/signaling pathway to permit its replication in host cells. Multiple proteins encoded by SARS-CoV-2, including ORF3, ORF6, M, and several non-structure proteins, inhibit RLR-induced IFN activation.^6–8^ Moreover, SARS-CoV-2 papain-like protease and M protein were shown to suppress MDA5 and MAVS activity, respectively.^9–11^ Intriguingly, SARS-CoV-2 proteins such as ORF9b, ORF3a, and 3CL, were reported to inhibit the cGAS-STING pathway.^12,13^ These viral antagonistic mechanisms may account for the dampened type-I IFN (IFN-I) levels in some severe COVID-19 cases.^14^ Despite these inhibitory machineries exploited by the virus, overt IFN activation and inflammatory responses were still detected in SARS-CoV-2-infected animals^15,16^ and peripheral blood or respiratory tract samples from COVID-19 patients.^17–21^ A major gap in our knowledge is how SARS-CoV-2 infection triggers host innate immune responses.

Here we report that the cytosolic DNA sensing cGAS-STING pathway is involved in IFNs production upon SARS-CoV-2 infection. The activation of cGAS relies on its recognition of cytoplasmic chromatin DNA generated upon cell-to-cell fusion, a widespread phenomenon in SARS-CoV-2-infected cells,^22^ organoids,^23^ animals,^24^ and in COVID-19 patients.^25^ Our study revealed a mechanism, distinct from the classical PAMP-PRR paradigm, by which the host innate immune system responds to viral infection by producing “danger signals” using self-materials.

## Results

### SARS-CoV-2 infection activates the cGAS-STING pathway

**Fig. 1:**
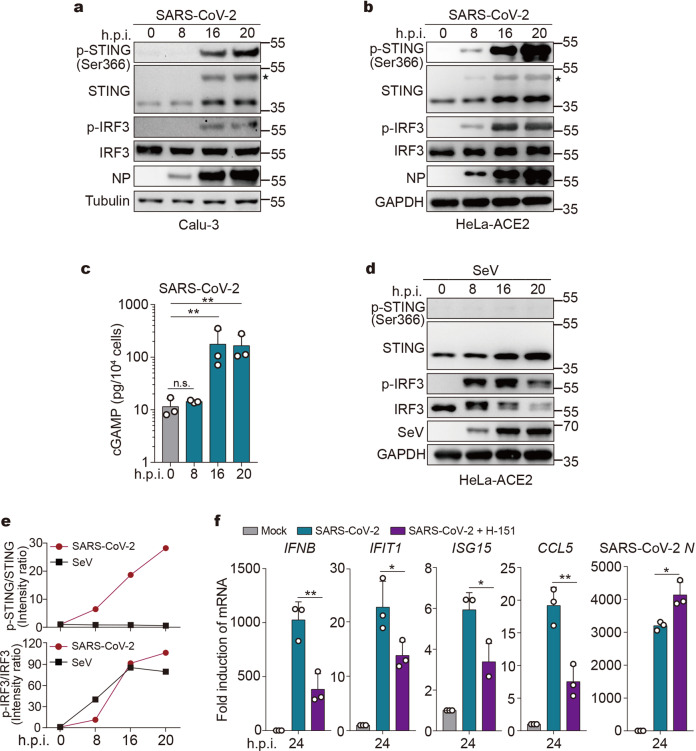
SARS-CoV-2 infection activates the cGAS-STING pathway. **a**, **b** Representative Western blots of STING and IRF3 phosphorylation upon SARS-CoV-2 infection (*n* = 2 independent experiments). Calu-3 (**a**) or HeLa-ACE2 (**b**) cells were mock-infected or infected with SARS-CoV-2 at an MOI of 0.5. At indicated time points after infection, cells were harvested and lysed. Lysates were then subjected to Western blot analysis using indicated antibodies. ✶Activated STING. **c** 2′3′-cGAMP levels in cells infected with SARS-CoV-2. HeLa-ACE2 were infected with SARS-CoV-2 as described in **b**. A competitive ELISA assay determined the 2′3′-cGAMP concentrations. Mean ± s.d., *n* = 3 independent experiments. **P < 0.01, n.s. not significant. Two-tailed Student’s *t*-test on log-transformed data. **d** STING and IRF3 phosphorylation upon SeV infection. HeLa-ACE2 cells were mock-infected or infected with SeV. Cells were harvested at indicated times and analyzed by Western blot. **e** Semi-quantitative analysis on Western blot results from **a** and **d** by densitometry. **f** Calu-3 cells were treated with DMSO or 200 ng/ml of H-151. After 2 h, cells were mock-infected or infected with SARS-CoV-2 at an MOI of 0.5 for 24 h. RNA extracted from the cells was evaluated by quantitative PCR. The data are expressed as fold change of the *IFNB*, *IFIT1*, *ISG15*, *CCL5*, and SARS-CoV-2 *N* mRNA levels relative to the *GAPDH* control. Mean ± s.d., *n* = 3 independent experiments. *P < 0.05, **P < 0.01, two-tailed Student’s *t*-test

We recently reported that SARS-CoV-2 infection failed to induce rapid IFN-β production in cultured cells,^6^ consistent with multiple inhibitory mechanisms against the RLR pathway imposed by the virus. Mysteriously, however, SARS-CoV-2 induces substantial IFN-β production at later time points of infection.^6^ These observations prompted us to hypothesize that a signaling pathway distinct from the RLR signaling is activated. Among the major host innate immune mechanisms that stimulate IFNs production is the cGAS-STING cytosolic DNA sensing pathway. Thus, we asked whether SARS-CoV-2 infection activates cGAS and STING. We infected human lung epithelial cell line Calu-3 and HeLa cells expressing ACE2 (HeLa-ACE2) with SARS-CoV-2, and then examined the phosphorylation of STING at Ser366 (phospho-STING^Ser366^), a hallmark of STING activation.^26^ At 8 h post infection, phospho-STING^Ser366^ was barely detected in Calu-3 and HeLa-ACE2 cells, while after infection for 16 h, both cell lines showed robust STING^Ser366^ phosphorylation (Fig. 1a, b). In line with this, cyclic dinucleotide 2′3′-cGAMP, a molecule produced by activated cGAS, was significantly increased after 16 h of infection (Fig. 1c). Hence, the cGAS-cGAMP-STING cascade is activated during SARS-CoV-2 infection.

As a comparison, we examined whether other RNA viruses can activate STING. In contrast to SARS-CoV-2, Sendai virus (SeV), a well-characterized agonist of the RLR signaling pathway, failed to stimulate STING ^Ser366^ phosphorylation despite its competent viral replication (Fig. 1d, e). Of note, at 8 h post infection, SeV substantially stimulated phosphorylation of IRF3 (Fig. 1d, e), a downstream protein for both RLR-MAVS and cGAS-STING pathways. In contrast, IRF3 phosphorylation was weakly stimulated after SARS-CoV-2 infection at 8 h, while it was sharply increased after infection at 16 h (Fig. 1a, b, e); the kinetic of IRF3 phosphorylation is consistent with that of STING phosphorylation. These observations suggest that SARS-CoV-2 counteracts RLR detection/signaling upon an early phase of infection, but effectively stimulates the cGAS-STING pathway upon a late phase of infection.

We subsequently asked whether STING-mediated signaling is required for SARS-CoV-2-triggered IFN responses. Although SARS-CoV-2 infection into Calu-3 cells induced the expression of IFN and IFN-stimulated genes (ISGs), including *IFNB*, *IFIT1*, *ISG15*, and *CCL5*, pharmacological inhibition of STING by an established compound, H-151,^27^ significantly decreased SARS-CoV-2-triggered IFN responses while increased virus replication (Fig. 1f). Hence, STING is not only activated but is also required for IFN production upon SARS-CoV-2 infection.

### cGAS colocalizes with cytosolic genomic DNA in SARS-CoV-2-induced syncytia

We next sought to determine how SARS-CoV-2 infection activates the cGAS-STING pathway. The genetic entity of SARS-CoV-2 is RNA, which does not stimulate cGAS. Recent studies suggested that cGAS can detect self-DNA presented in the cytosol, including chromosomal DNA^28–31^ and mitochondrial DNA.^32^ We thus hypothesized that cGAS is activated by self-DNA upon SARS-CoV-2 infection. Using fluorescent microscopy, we found that a prominent cellular morphology feature in response to SARS-CoV-2 infection is cell–cell fusion and the formation of syncytia (a single cell containing several nuclei) (Supplementary Fig. 1a). SARS-CoV-2 enters host cells by membrane fusion dependent on the interaction between viral spike protein and cell surface protein ACE2.^33^ During subsequent viral replication, the infected cells can form syncytia mediated by cell surface expression of the spike protein.^22^ We found that, at 20 h post infection, substantial HeLa-ACE2 cells were fused to form multinucleated cells (Supplementary Fig. 1b). SARS-CoV-2-induced cell fusion was further observed in other cell types such as THP-1 macrophages (Supplementary Fig. 1c). Further, infection of K18-hACE2 transgenic mice with SARS-CoV-2 resulted in positive staining of viral NP protein and signs of perivascular inflammation, along with the presence of multinucleated syncytial cells (Supplementary Fig. 1d), indicating that SARS-CoV-2 induces syncytia formation in vivo.

**Fig. 2:**
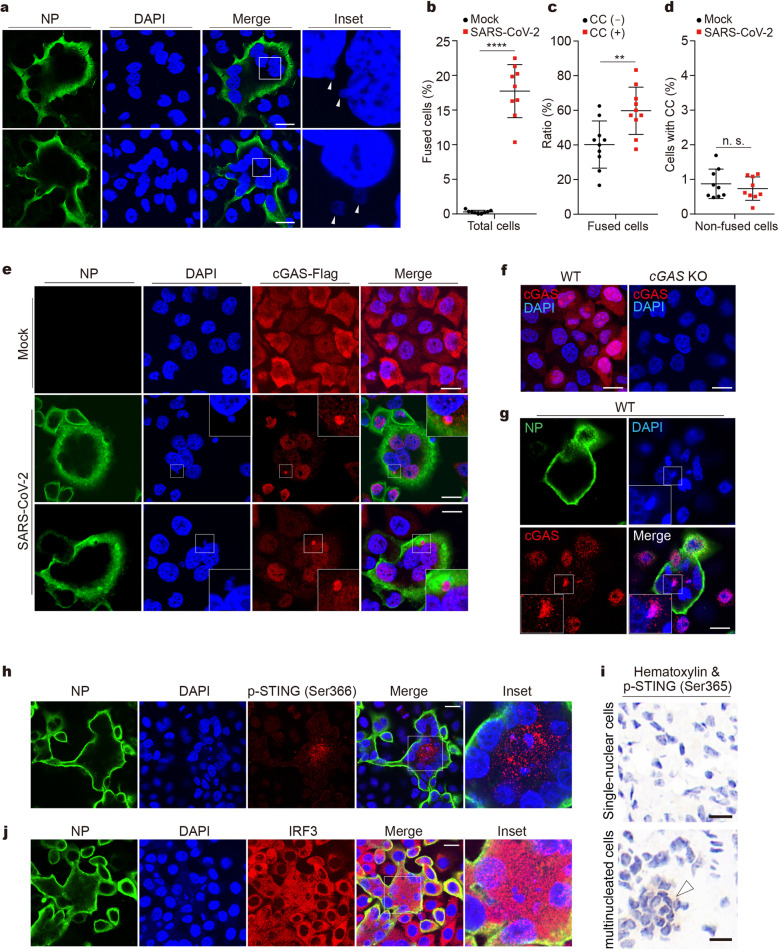
cGAS colocalizes with cytosolic genomic DNA in SARS-CoV-2-induced syncytia. **a** Representative confocal immunofluorescence images of SARS-CoV-2-induced syncytia. HeLa-ACE2 cells were infected with SARS-CoV-2 at an MOI of 0.5. After 18 h, cells were fixed and stained for DNA with DAPI (blue) and viral nucleocapsid protein (NP) with anti-NP antibody (green). Triangles indicate budding chromatin (upper) or cytosolic chromatin (lower). Scale bar, 20 μm. **b**–**d** Quantification of cells for parameters as indicated. Cells were quantified for three different fields with at least 400 cells. Mean ± s.d., Data were pooled from 3 independent experiments. ****P < 0.0001, **P < 0.01, two-tailed Student’s *t*-test. CC cytoplasmic chromatin. **e** Representative images of cells stained for NP, DNA, and cGAS-Flag. cGAS-null HeLa-ACE2 cells reconstituted with cGAS-Flag were infected with SARS-CoV-2 at an MOI of 0.5 for 18 h, followed by staining with anti-NP antibody (green), DAPI (blue), and anti-Flag antibody (red) as indicated. Scale bar, 20 μm. **f** Cellular distribution of endogenous cGAS. HeLa-ACE2 cells (left) or cGAS-null HeLa-ACE2 cells (right) were stained with anti-cGAS antibody (red) and DAPI (blue). Scale bar, 20 μm. **g** HeLa-ACE2 cells were infected with SARS-CoV-2, followed by staining with anti-NP antibody (green), DAPI (blue), and anti-cGAS antibody (red) as indicated. Scale bar, 20 μm. **h** HeLa-ACE2 cells were treated as described in **e** and were stained with anti-NP antibody (green), DAPI (blue), and anti-phospho-STING (Ser366) antibody (red). Scale bar, 20 μm. **i** K18-hACE2 transgenic mice were infected with 10^5^ TCID_50_ of SARS-CoV-2 for 3 days. Mouse lungs were harvested and subjected to immunohistochemistry analysis with anti-phospho-STING (Ser365) antibody (diaminobenzidine visualization). Nuclei were stained with hematoxylin (dark blue). Scale bar, 10 μm. **j** HeLa-ACE2 cells were treated as described in **e** and were stained with anti-NP antibody (green), DAPI (blue), and anti-IRF3 antibody (red). Scale bar, 20 μm

Upon staining of DNA with DAPI, we observed nuclear membrane blebbing (Fig. 2a, upper) accompanied by budding off nuclei (Fig. 2a, lower) in the fused HeLa-ACE2 cells and human airway Calu-3 cells (Supplementary Fig. 2a). The frequency of cell fusion and cytoplasmic DAPI events were then quantified. Approximately 18% of the cells were fused after SARS-CoV-2 infection (Fig. 2b). Cytoplasmic DAPI events are mainly observed in fused cells (Fig. 2c) but are rarely found in non-fused cells (Fig. 2d). Moreover, SARS-CoV-2 infection does not increase the production of cytoplasmic chromatin in non-fused cells (Fig. 2d). We also found that higher infection dose and cell confluency promotes cytoplasmic chromatin formation (Supplementary Fig. 2b). These data revealed that SARS-CoV-2-induced cell fusion promotes the generation of cytoplasmic genomic DNA.

The nuclear membrane blebbing and nucleus-to-cytoplasm trafficking of genomic DNA observed upon SARS-CoV-2 infection are reminiscent of cytoplasmic chromatin in cellular senescence. In senescent cells, fragments of chromatin are shuttled to the cytoplasm, via nuclear membrane blebbing,^34^ and are recognized by cGAS.^30,31,35^ We and others reported that cytoplasmic chromatin activates the cGAS-STING pathway, stimulating the pro-inflammatory responses of senescence.^30,31,35^ In addition to senescence, cytoplasmic chromatin is observed in cancer, aging, and other stressed conditions, leading to cGAS activation.^36,37^ We therefore hypothesized that cytoplasmic chromatin upon SARS-CoV-2 infection activates cGAS.

We subsequently examined the localization of cGAS. cGAS^−/−^ HeLa-ACE2 cells reconstituted with cGAS-Flag were stained with an anti-Flag antibody. In non-infected cells, cGAS showed a diffuse distribution in both cytoplasm and nucleus (Fig. 2e). Upon SARS-CoV-2 infection, in fused cells staining positive for NP, cGAS displayed nuclear distribution and aggregated to form puncta that colocalize with cytoplasmic chromatin (Fig. 2e and Supplementary Fig. 2c); cGAS did not form cytoplasmic aggregation in non-fused cells (Fig. 2e and Supplementary Fig. 2c). These observations were fully recapitulated by staining endogenous cGAS with an anti-cGAS antibody in wildtype HeLa-ACE2 cells (Fig. 2f, g). The colocalization between cytoplasmic chromatin and cGAS is consistent with the generation of cGAMP in infected cells (Fig. 1c). Further, STING is phosphorylated (Fig. 2h) and redistributed from the endoplasmic reticulum to confined compartments in fused cells (Supplementary Fig. 2d), typical for STING activation. Moreover, immunohistochemistry analysis of lungs from SARS-CoV-2-infected mice showed specific signals of STING phosphorylation in the multinucleated cells by staining with the mouse-specific phospho-STING^Ser365^ antibody (Fig. 2i, indicated by the triangle), suggesting that STING is activated upon syncytia formation during viral infection in vivo. Finally, IRF3 activation, indicated by cytoplasm-to-nucleus translocation of IRF3, is also observed in fused cells (Fig. 2j). Taken together, these results indicate that cytoplasmic genomic DNA formed in syncytia activates the cGAS-STING pathway upon SARS-CoV-2 infection.

### Cell fusion in the absence of viral infection activates the cGAS-STING pathway

**Fig. 3:**
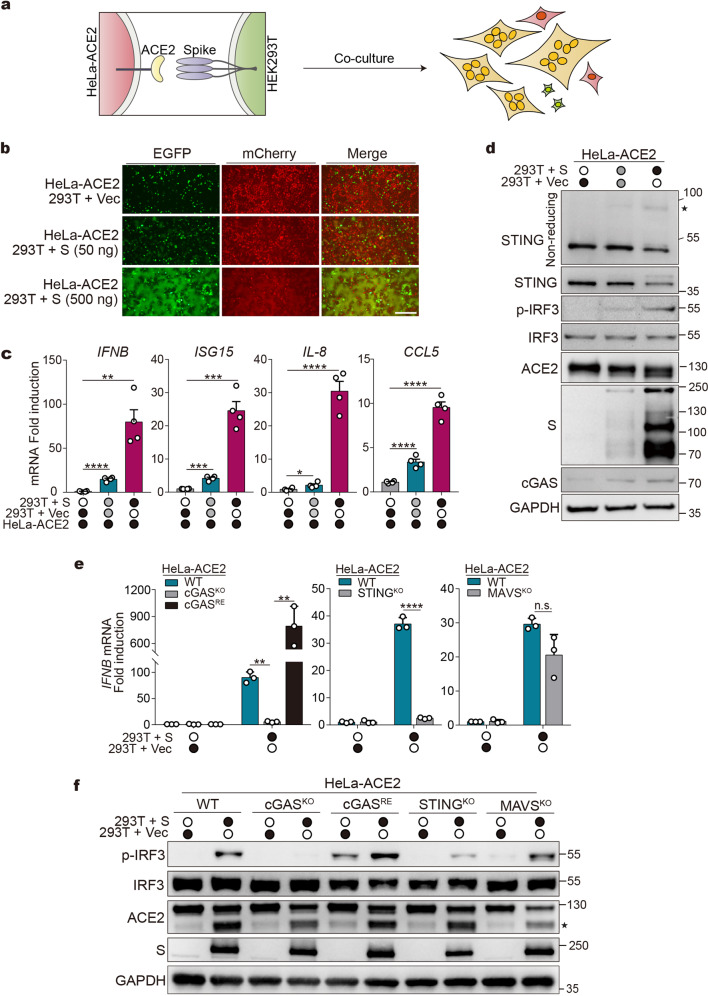
Cell fusion activates the innate immune response via the cGAS-STING pathway. **a** Scheme of co-culture experiment. **b** Representative fluorescence images of co-culture experiment. HEK293T cells were transfected with vector control (Vec) or increasing amounts of plasmids expressing spike (S), along with plasmids expressing EGFP for 24 h. Cells were then detached and mixed with HeLa-ACE2 expressing mCherry (HeLa-ACE2-mCherry). After 8 h, cells were subjected to fluorescence microscopy analysis. Scale bar, 250 μm. **c** Cytokine genes/ISGs expression in co-cultured cells. Cells were co-cultured as indicated in **b**. RNA extracted from the cells was evaluated by quantitative PCR. The data are expressed as fold change of the *IFNB*, *ISG15*, *IL8*, and *CCL5* mRNA levels relative to the *GAPDH* control. Mean ± s.d., *n* = 4 independent experiments. **P < 0.01, ***P < 0.001, ****P < 0.0001, two-tailed Student’s *t*-test. **d** Western blot analysis of cells from co-culture experiment as described in **b** using indicated antibodies. STING blots were performed under non-reducing (top) or reducing conditions. ✶STING dimer. **e** HEK293T cells were transfected with vector control (Vec) or plasmids expressing spike. After 24 h, cells were detached and mixed with HeLa-ACE2-mCherry cells as indicated. cGAS^KO^, STING^KO^, and MAVS^KO^ represent cells depleted of indicated genes. cGAS^RE^ represents cGAS-null cells re-expressed cGAS. The expression of *IFNB* mRNA was assayed as described in **c**. Mean ± s.d., *n* = 3 independent experiments. **P < 0.01, ****P < 0.0001, n.s. not significant. two-tailed Student’s *t*-test. **f** Western blot analysis of cells from the co-culture experiment as described in **e**. ✶ACE2 fragments generated during cell co-culture. S spike

We next asked whether cell fusion per se can provoke antiviral responses in the absence of viral infection. To address this, we developed a co-culture system to recapitulate SARS-CoV-2-induced cell fusion. HEK293T cells were transfected with plasmids expressing EGFP and spike protein of SARS-CoV-2 and were mixed with HeLa-ACE2 cells expressing mCherry (illustrated in Fig. 3a). As measured by the amounts of EGFP/mCherry double-positive cells, we found that spike protein stimulates cell fusion in a dose-dependent manner (Fig. 3b). We then examined the expression of genes involved in antiviral responses. Cell fusion per se mediated by spike and ACE2 potently induced the expression of cytokines/ISGs, including *IFNB*, *ISG15*, *IL8*, and *CCL5* (Fig. 3c). The induction of antiviral genes is dependent on cell fusion rather than spike expression, because co-culture of HEK293T cells expressing spike protein (HEK293T(S)) with wildtype HeLa cells that do not express ACE2 (Supplementary Fig. 3a) failed to trigger cell fusion (Supplementary Fig. 3b) and cytokines/ISGs expression (Supplementary Fig. 3c). Thus, cell fusion mediated by spike and ACE2 activates innate immune response in the absence of viral infection.

We subsequently examined the status of cGAS and STING in the co-culture system. We found that the co-culture of HEK293T(S) cells with HeLa-ACE2 cells, rather than wildtype HeLa cells, activated STING, as detected by the formation of STING homodimers, the active form of STING (Fig. 3d and Supplementary Fig. 3d). Concomitantly, cell fusion resulted in IRF3 phosphorylation (Fig. 3d and Supplementary Fig. 3d), suggesting the activation of the STING-IRF3 signaling axis. Moreover, pretreatment of HEK293T(S) cells with a neutralizing antibody against spike protein markedly impeded cell fusion (Supplementary Fig. 3e) and STING-IRF3 activation (Supplementary Fig. 3f) in the co-culture assay, corroborating that cell fusion per se stimulates STING-IRF3. We further examined whether cGAS and STING are required for cell fusion-triggered antiviral signaling. In the co-culture system, genetic ablation of cGAS in HeLa-ACE2 cells (HeLa-cGAS^KO^-ACE2, Supplementary Fig. 3g) suppressed *IFNB* induction (Fig. 3e) and IRF3 phosphorylation (Fig. 3f) without affecting cell fusion (Supplementary Fig. 3h). Re-expression of cGAS in cGAS-null cells (HeLa-cGAS^RE^-ACE2, Supplementary Fig. 3g) rescued *IFNB* expression (Fig. 3e) and IRF3 phosphorylation (Fig. 3f). Genetic depletion of STING (Supplementary Fig. 3i) phenocopied the effect of cGAS (Fig. 3e, f). In contrast, MAVS, the adaptor protein critical for RLR-mediated signaling, is dispensable for cell fusion-induced IFN activation (Supplementary Fig. 3j and Fig. 3e, f). Therefore, we conclude that the cGAS-STING pathway is required for cell fusion-induced IFN and pro-inflammatory responses.

### Sensing of cytoplasmic chromatin by cGAS in syncytial cells

**Fig. 4:**
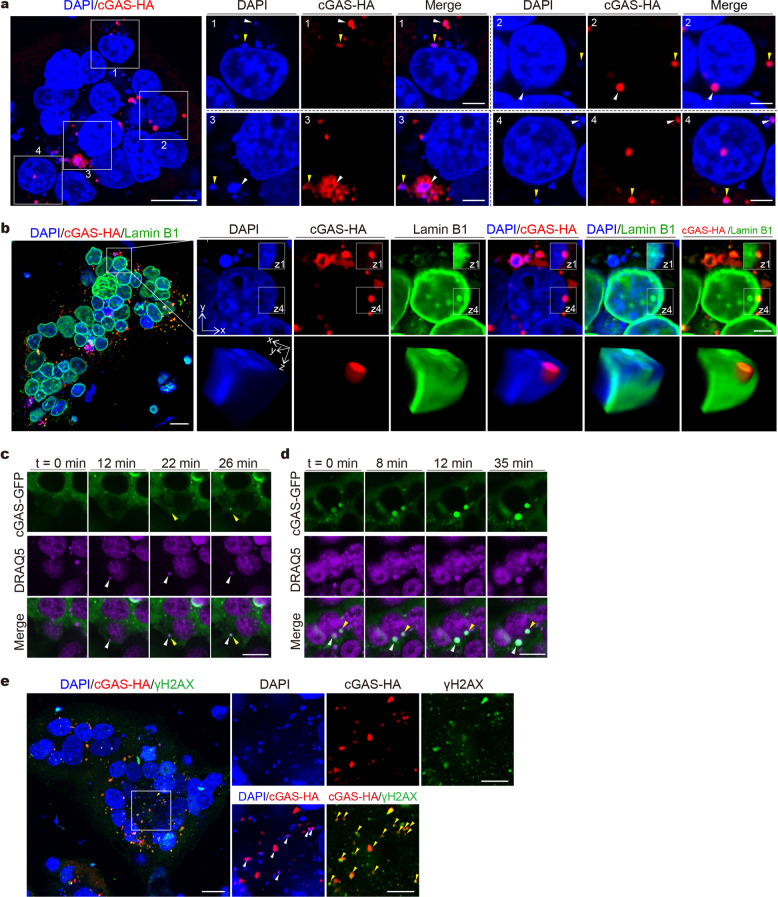
cGAS is colocalized with cytoplasmic chromatin in syncytial cells. **a** Representative confocal immunofluorescence images of co-cultured cells stained for DNA and cGAS-HA. HEK293T cells transfected with plasmids expressing spike (HEK293T(S)) were co-cultured with cGAS-null HeLa-ACE2 cells transfected with cGAS-HA. After 8 h, cells were stained with DAPI (blue) and anti-HA (red) antibody as indicated. Triangles indicate colocalization of cGAS with cytosolic chromatin. **b** Representative confocal immunofluorescence images of co-cultured cells stained for DNA, cGAS-HA, and Lamin B1. Co-culture experiments were performed as described in **a**. Cells were stained with DAPI (blue), anti-HA (red), and anti-Lamin B1 (green) antibodies. Three-dimensional reconstructed images based on z-stack images were displayed as volume view. **c**, **d** Extracted frames from live-cell imaging of co-cultured cells using confocal microscopy. HEK293T(S) cells transfected with plasmids expressing cGAS-GFP were co-cultured with cGAS-null HeLa-ACE2 cells transfected with plasmids expressing cGAS-GFP. After 1 h, cells were stained with DRAQ5 (purple) for visualizing DNA and subjected to live-cell imaging. **e** Co-culture experiments were performed as described in **a**. Cells were stained with DAPI (blue), anti-HA (red) antibody, and anti-γH2AX (green) antibody. Scale bars, 20 μm or 5 μm (inset)

We examined the mechanisms for cGAS activation in syncytial cells in the absence of infection, and hypothesized that cytoplasmic chromatin activates cGAS. We co-cultured HeLa-ACE2 cells expressing cGAS-HA with HEK293T(S) cells, followed by cGAS-HA and DNA co-staining. Using confocal fluorescent microscopy, we found that multiple DNA-containing puncta appeared in the perinuclear region and are partially colocalized with cGAS in the fused cells (Fig. 4a). In some cases, cGAS colocalized with nuclear DNA that is in the process of budding off the nucleus (Fig. 4a, Inset 2). To dissect this process in more depth, we generated three-dimensional reconstructed images, co-staining for Lamin B1, cGAS, and DNA. This analysis showed that a hemispheric body containing cGAS and chromosomal DNA is localized at the fringe of the nucleus and is beneath or in the process of penetrating the nuclear lamina meshwork, representing a typical nuclear membrane bleb (Fig. 4b). We further performed live-cell imaging to document this dynamic process. HeLa-ACE2 cells expressing cGAS-GFP were co-cultured with HEK293T cells expressing cGAS-GFP and spike, and were stained with DRAQ5 for live-cell imaging of DNA. This experiment revealed that cell fusion leads to the formation of nuclear membrane blebs accompanied by subsequent budding off to form cytoplasmic chromatin (Fig. 4c, d). In some cases, cGAS was recruited to cytoplasmic chromatin after nuclear membrane blebs budded off nuclei (Fig. 4c and Supplementary Movie 1). In other cases, cGAS was recruited to intra-nuclear chromatin that is in the process of nucleus-to-cytoplasm trafficking and was co-shuttled to the cytoplasm (Fig. 4d and Supplementary Movie 2).

Because cytoplasmic chromatin in the context of senescence is associated with DNA damage response,^31^ we next examined if cell fusion-induced cytoplasmic chromatin stains positive for DNA damage markers. By staining co-cultured cells for DNA damage marker γH2AX, we found that cGAS strongly co-colocalizes with γH2AX puncta in the cytoplasm and occasionally in the nucleus (Fig. 4e and Supplementary Fig. 4). Three-dimensional reconstructed images, focusing on nuclear events, showed that γH2AX colocalizes with cGAS at the budding chromatin undergoing nucleus-to-cytoplasm transport (Supplementary Fig. 4). In sum, we reported that cytoplasmic chromatin is sensed by cGAS in syncytia mediated by spike and ACE2.

### Syncytia formation disrupts actin cytoskeleton and nucleoskeleton

**Fig. 5:**
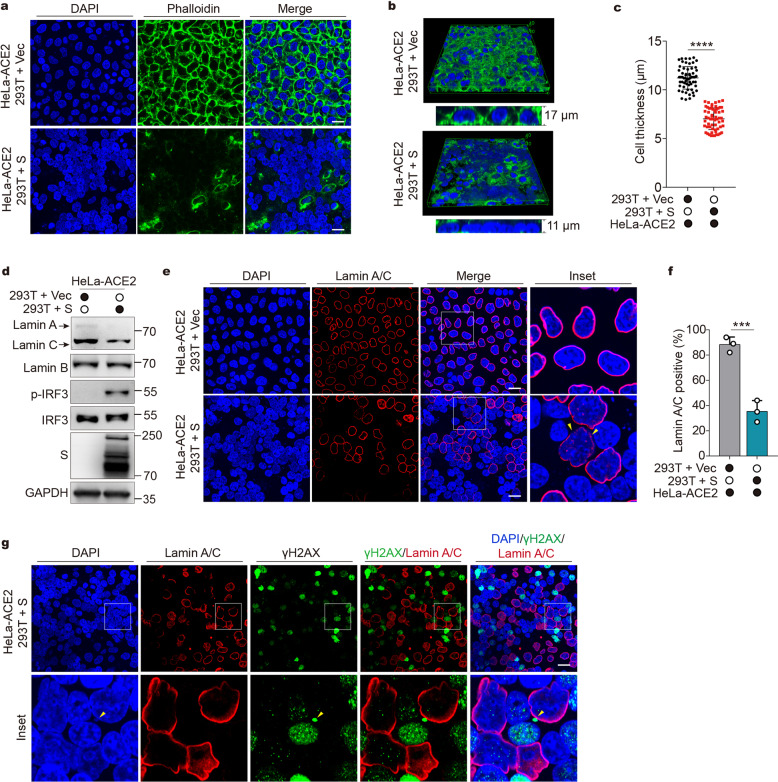
Syncytia formation disrupts actin cytoskeleton and nucleoskeleton. **a** Representative fluorescence images of co-culture experiment. HEK293T cells were transfected with vector control (Vec) or plasmids expressing spike (S) for 24 h. Cells were then detached and mixed with HeLa-ACE2. After 4.5 h, cells were stained with DAPI (blue) and fluorescently labeled phalloidin (green) as indicated. Scale bar, 20 μm. **b** Three-dimensional reconstructed images of **a** were displayed as volume view. **c** Quantification of cell thickness. Cells were measured for height based on z-stack images. Mean ± s.d. Data were pooled from 3 independent experiments. ****P < 0.0001, two-tailed Student’s *t*-test. **d** Cells were treated as described in **a** and subjected to Western blot analysis using indicated antibodies. **e** Cells were treated as described in **a** and stained with DAPI (blue) and anti-lamin A/C antibody (red) as indicated. Scale bar, 20 μm. **f** Quantification of Lamin A/C positive cells from experiments described in **e**. Mean ± s.d., *n* = 3 independent experiments. ***P < 0.001. Two-tailed Student’s *t*-test. **g** HEK293T cells were transfected with plasmids expressing S for 24 h. Cells were then detached and mixed with HeLa-ACE2. After 4.5 h, cells were stained with DAPI (blue), anti-γH2AX (green) antibody, and anti- Lamin A/C antibody (red) as indicated. Scale bar, 20 μm

We next explored the possible mechanisms by which syncytia formation induces cytoplasmic chromatin. The most noticeable feature observed in the syncytial cells is the large aggregation of the nuclei accompanied by frequent nuclear blebbing. Because nuclear positioning is governed by the cytoskeletal elements such as actin filaments (F-actin),^38^ we first examined F-actin organization during cell fusion. HeLa-ACE2 cells were co-cultured with HEK293T or HEK293T (S) to yield non-fused controls and fused cells, respectively, followed by staining with fluorescently labeled phalloidin, a peptide that specifically binds actin filaments. F-actin staining revealed normal filamentous actin structures in non-fused cells (Fig. 5a, upper). However, F-actin signals are largely diminished in fused cells (Fig. 5a, lower), suggesting that actin filaments collapsed during cell fusion. Furthermore, three-dimensional reconstruction analysis demonstrated that the nuclei of fused cells were deformed, as evidenced by significantly reduced nucleus thickness (Fig. 5b, c). These results suggested that syncytia formation could disrupt the actin cytoskeleton, allowing mechanical constraints on the nucleus to be released, and thus lead to nuclear malpositioning and deformation.

Because decreased cytoskeletal tension could lower the amount of nuclear envelope Lamin A and C proteins,^39^ which play pivotal roles in maintaining lamina integrity and nuclear morphology, we examined lamin A/C expression in co-cultured cells. Indeed, Lamin A/C protein levels were substantially reduced in fused cells compared to those in non-fused cells (Fig. 5d). In contrast, Lamin B level remained unchanged (Fig. 5d). By confocal microscopy, Lamin A/C were found in the majority of non-fused cells, localizing at the nuclear envelope (Fig. 5e, upper, and Fig. 5f), whereas in fused cells, only a subset of nuclei were positive for Lamin A/C (Fig. 5e, lower, and Fig. 5f), and fragmented Lamin A/C structure was observed (Fig. 5e, indicated by triangles), suggesting a breakdown of Lamin A/C-associated lamina meshwork. Again, no reduction of Lamin B expression was found in fused cells by microscopy (Supplementary Fig. 5). These observations are consistent with the fact that Lamin A/C, rather than Lamin B, respond to cell mechanical changes.^40^

Because Lamin A/C defect or depletion results in DNA damage, nuclear blebbing, and micronuclei generation,^41–43^ we reason that Lamin A/C disruption in response to cell fusion could reduce genome stability and contribute to cytoplasmic chromatin formation. Indeed, in fused cells, γH2AX foci largely accumulated in the nuclei lacking Lamin A/C, whereas Lamin A/C-positive nuclei exhibited far fewer and weaker γH2AX signals (Fig. 5g). Moreover, chromatin fragments were found colocalized with γH2AX puncta in the cytoplasm (Fig. 5g, indicated by triangles), likely shed by γH2AX-positive and Lamin A/C-negative nuclei. Thus, nuclear Lamin A/C dysfunction may underly cytoplasmic chromatin formation in the context of cell fusion.

### Targeting cGAS-STING pathway as potential therapeutics against SARS-CoV-2

**Fig. 6:**
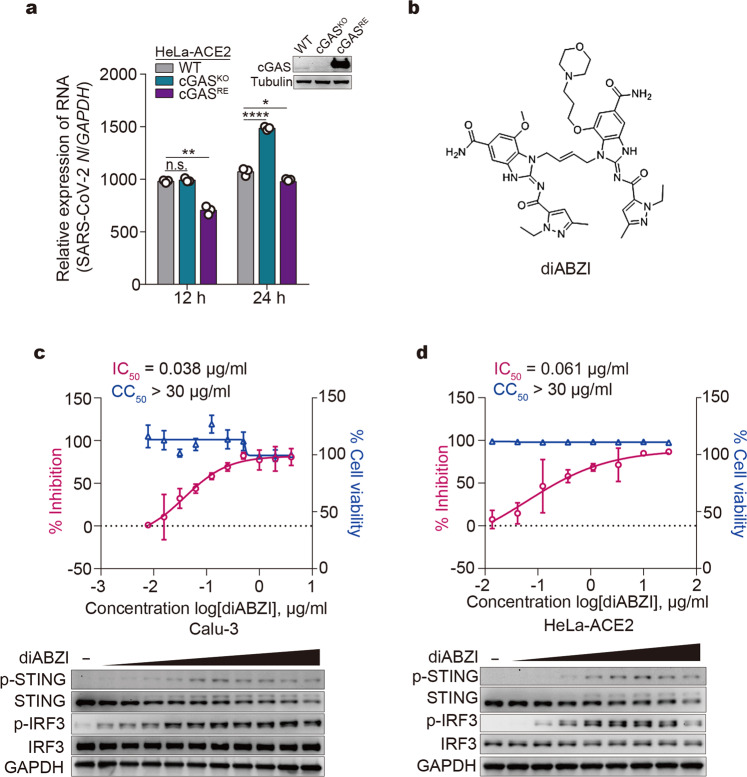
Targeting cGAS-STING pathway as potential therapeutics against SARS-CoV-2. **a** Effect of cGAS expression on SARS-CoV-2 replication. Wildtype HeLa-ACE2 (WT), HeLa-cGAS^KO^-ACE2, and HeLa-cGAS^RE^-ACE2 cells were infected with SARS-CoV-2 at an MOI of 0.5. At indicated times, total RNA extracted from cells was evaluated by quantitative PCR. The data are expressed as fold changes of the RNA levels of the viral *N* gene relative to the *GAPDH* control. Mean ± s.d., *n* = 3. *P < 0.05, **P < 0.01, ****P < 0.0001, n.s. not significant. Two-tailed Student’s *t*-test. **b** The chemical structure of diABZI. **c**, **d** Antiviral effect of diABZI on SARS-CoV-2. Calu-3 (**c**) or HeLa-ACE2 (**d**) cells were treated with serially diluted diABZI for 24 h (Calu-3) or 1 h (HeLa-ACE2). Cells were then subjected to viability assay or infected with SARS-CoV-2 at an MOI of 0.2. After 24 h (Calu-3) or 48 h (HeLa-ACE2), supernatants were harvested for RNA extraction, followed by absolute quantification of viral N mRNA by PCR. Mean ± s.d., *n* = 4. The IC_50_ (the half-maximal inhibitory concentration) and CC_50_ (the half-maximal cytotoxic concentration) values were calculated using Prism software. Lower panels showed diABZI-induced STING and IRF3 activation. Calu-3 (**c**) or HeLa-ACE2 (**d**) cells were treated with serially diluted diABZI for 24 h and 6 h, respectively, followed by Western blot analysis using indicated antibodies

Lastly, we investigated the role of the cGAS-STING pathway in restraining SARS-CoV-2 replication in host cells. HeLa-ACE2, HeLa-cGAS^KO^-ACE2, and HeLa-cGAS^RE^-ACE2 cells were infected with SARS-CoV-2. At 12 h post infection, depletion of cGAS did not affect virus replication, while re-expression of cGAS in cGAS-null cells showed antiviral effects (Fig. 6a), possibly due to overexpression of cGAS (Fig. 6a). At 24 h post infection, cGAS depletion led to significantly elevated viral replication (Fig. 6a), indicating that cGAS-mediated signaling is required for optimal host defense against SARS-CoV-2. The antiviral role for cGAS at a later time point coincides with the timeframe required for cell-to-cell fusion, which occurs around 12–14 h post infection.

Recently, STING agonists were pre-clinically or clinically tested as antitumor drugs due to their immunostimulatory effects.^44^ We therefore examined whether STING agonists can be harnessed as antiviral drugs against SARS-CoV-2. diABZI, a recently developed synthetic non-nucleotide STING agonist^45^ (Fig. 6b), exerts strong STING-stimulating activity and potent antiviral efficacy against SARS-CoV-2 with half-maximal inhibitory concentration (IC_50_) values of 0.038 μg/ml (≈0.045 μM) in Calu-3 cells (Fig. 6c) and 0.061 μg/ml (≈0.072 μM) in HeLa-ACE2 cells (Fig. 6d). This diABZI-mediated antiviral efficacy is comparable to that of Remdesivir (IC50 = 0.054 μM in Calu-3 and 0.024 μM in HeLa-ACE2) (Supplementary Fig. 6a, b), a direct-acting antiviral drug against SARS-CoV-2. Moreover, SR-717, a novel cGAMP mimetic that activates STING,^46^ also inhibited SARS-CoV-2 replication (Supplementary Fig. 6c). Taken together, these results strongly suggest an antiviral role for the cGAS-STING pathway in restraining SARS-CoV-2.

## Discussion

Our study revealed that host self-DNA, distinct from pathogen genetic molecules, can serve as a danger signal to trigger antiviral responses in SARS-CoV-2 infection. This response relies on SARS-CoV-2-induced syncytia, which causes the nuclear export of chromosomal DNA that activates the cytosolic DNA sensing cGAS-STING pathway. This finding expands the horizon of how innate immune system reacts to pathogen infection, beyond the established PAMP-PRR model. Because viruses possess multiple mechanisms to inhibit PAMP-PRR sensing/signaling, the host cells have evolved a mechanism that produces danger signals using self-constituents. This mechanism offers additional routes to permit communication with neighboring cells and the immune system upon viral infection.

Cell fusion can be observed during infection by a panel of enveloped viruses.^47,48^ This process has been considered as a route leveraged by viruses for their cell-to-cell spreading.^49^ Our data showed that the host exploits cell fusion to restrain virus replication, suggesting that cell fusion could be an interface of virus–host mutual antagonism. Besides viral infection, cell fusion is involved in other biological processes such as embryogenesis, tissue morphogenesis,^50^ and cancer.^51^ In addition, ectopic expression of proteins that drive cell fusion promotes senescence of primary cells associated with pro-inflammatory responses.^52^ A commonality of these pathophysiological conditions is the secretion of growth factors/cytokines. It is thus valuable to investigate whether the formation of cytoplasmic chromatin is involved in these cell fusion events.

Chromosomal DNA in the cytoplasm can be generated by chromosome mis-segregation during cell division, such as micronuclei,^28,29^ or by nucleus-to-cytoplasm transport of chromatin fragments under certain pathological conditions such as cellular senescence.^30,31^ In SARS-CoV-2-induced syncytia, no cell division was observed under live-cell imaging experiments, and hence the cytoplasmic chromosomal DNA does not fit into the criteria of micronuclei. Instead, the nuclear membrane blebbing and subsequent partition into the cytoplasm in SARS-CoV-2 infected cells are akin to the generation of cytoplasmic chromatin fragments (CCFs) in senescence.^34^ CCFs in senescence stain positive for repressive heterochromatin marks and negative for active euchromatin marks and are positive for γH2AX,^34^ indicating that CCFs are derived from fragments of chromatin and are associated with DNA damage response. While senescence is usually established in primary cells in days to weeks, cytoplasmic chromatin observed during SARS-CoV-2 infection occurs within hours and can be seen in transformed cancer cells, suggesting that the infected cells do not require senescence to induce pro-inflammatory responses. We showed that cell fusion rapidly disrupted cellular cytoskeleton and nucleoskeleton, as manifested by the breakdown of actin filaments and reduction in Lamin A/C level. Lamin A/C provides mechano-protection of the genome, and abnormalities in Lamin A/C cause profound nuclear defects, such as DNA damage, nuclear envelope rupture, and micronuclei formation.^41–43,53^ Thus, we reason that cell fusion-induced nuclear lamina dysfunction underlies the formation of cytoplasmic chromatin. However, how this process is triggered and regulated remains largely elusive and represents a future direction for research.

Recently, it has been reported that the RLR MDA5 plays a critical role in detecting SARS-CoV-2 infection.^54^ As SARS-CoV-2 robustly inhibits RLR-mediated IFN activation,^6^ the host could leverage the syncytia-associated cytoplasmic chromatin-cGAS-STING pathway to stimulate IFN responses to curb SARS-CoV-2 replication. Because SARS-CoV-2 rapidly replicates in cells^55^ and high SARS-CoV-2 loads were detected very soon after symptom onset in patients,^56^ it can be postulated that syncytia formation, which is dependent on spike proteins produced by viral replication, could occur shortly after infection and activate IFN at early disease stages. Moreover, we demonstrated that diABZI, a STING agonist, exerts potent antiviral effects against SARS-CoV-2. During the review process of our study, diABZI was reported to inhibit SARS-CoV-2 replication in vitro and in vivo.^57,58^ diABZI restricts virus replication by upregulating IFNs,^45^ which could inhibit virus at multiple steps, including viral entry and syncytia formation.^22^ Compared to bioproducts such as IFNs, STING agonists are more economical and thermally stable. Considering that SARS-CoV-2 possesses countermeasures to block IFN activity,^6^ and that STING can function via IFN-independent mechanisms such as translation inhibition^59^ and autophagy,^60^ we reason that STING agonist-based therapies may offer new opportunities to treat COVID-19.

On the other hand, heightened IFNs production is associated with severe COVID-19.^18^ Besides, cytoplasmic chromatin can trigger inflammation that contributes to tissue damage and disease progression.^31,61^ Thus, the chromatin-cGAS-IFN signaling axis needs to be tightly regulated to prevent immunopathological outcomes, especially at late disease stages. Moreover, syncytial cells can undergo lytic cell death such as pyroptosis,^22,62^ which results in the release of damage-associated molecular patterns (DAMPs) such as nucleic acids and ATP.^63^ These DAMPs could be recognized by neighboring cells, triggering the production of proinflammatory cytokines and chemokines. Therefore, syncytia formation and destruction may contribute to immunopathological outcomes such as hypercytokinemia in COVID-19 patients. In this scenario, inhibitors targeting cGAS-STING or cell fusion may suppress inflammation-associated disorders in COVID-19 patients. Recently, endeavors have been made to identify drugs that could block SARS-CoV-2-induced syncytia.^64^ Future studies are needed to unravel the disease stages of COVID-19 that are likely to be responsive to targeting the pathway we discovered herein.

## Materials and methods

HEK293T (ATCC, #CCL-11268), HeLa (ATCC, #CCL-2), Calu-3 (ATCC, #HTB-55), HeLa-ACE2, HeLa-cGAS^KO^-ACE2, HeLa-cGAS^RE^-ACE2, HeLa-STING^KO^-ACE2, and HeLa-MAVS^KO^-ACE2 cells were maintained in Dulbecco’s modified Eagle’s medium (Gibco) supplemented with 10% (vol/vol) FBS (Biological Industries) and antibiotics. The THP-1 cells (ATCC #TIB-202) were maintained in RPMI1640 (HyClone) supplemented with 10% (vol/vol) FBS (Gibco) and antibiotics. HeLa-ACE2 cells, HeLa-cGAS^KO^-ACE2 cells, and HeLa-cGAS^RE^-ACE2 cells were generated by lentiviral transduction of HeLa, HeLa-cGAS^KO^, and HeLa-cGAS^RE^ cells^65^ with ACE2. HeLa-STING^KO^-ACE2 cells and HeLa-MAVS^KO^-ACE2 cells were generated by lentiviral transduction of CRISPR-Cas9-engineered HeLa-STING^KO^ and HeLa-MAVS^KO^ cells with ACE2. SARS-CoV-2 virus infection was performed as described previously.^6^ All experiments with the SARS-CoV-2 virus were conducted in the BSL-3 laboratory.

### Lentivirus packaging and infection

The plasmid expressing ACE2 and mCherry, together with lentiviral packaging plasmids pVSVG and pR8.74 (Addgene), was transfected into HEK293T cells using the X-tremeGENE HP DNA transfection reagent (Roche, 06366546001) to produce the lentivirus. HeLa cells were infected with lentivirus at an MOI >1 for 72 h and were then subjected to FACS sorting to enrich mCherry positive cells.

### Co-culture experiment

HEK293T cells were plated 24 h before transfection at a density of 1.5 × 10^5^ cells per well in the 24-well plate and were transfected with SARS-CoV-2 spike expression plasmid. HeLa-ACE2 cells were seeded at a density of 1 × 10^5^ cells per well in the 24-well plate. Twenty-four h after transfection, 5 × 10^5^ HEK293T cells were seeded onto HeLa-ACE2 cells and co-cultured for the indicated period.

### Plasmids and reagents

SARS-CoV-2 spike (S) expression plasmid was described previously.^6^ The HA-tagged and GFP-tagged cGAS constructs were described previously.^66^ DRAQ5 (ab108410) was purchased from Abcam. DAPI (4′,6-diamidino-2-phenylindole) (40728ES03) was from YEASEN. SARS-CoV-2 Neutralizing Antibody (A19215) was from ABclonal. Alexa Fluor 488 Phalloidin (A12379) was from Thermo Scientific. STING-specific inhibitor H-151 (inh-h151) was from InvivoGen. diABZI STING agonist (compound 3) (S8796) and SR-717 lithium (S0853) were from Selleck.

### Immunoblotting

Cells were lysed, and an equal amount of each lysate was subsequently analyzed by SDS–PAGE following standard procedures. The antibodies used for immunoblotting were: rabbit-anti-cGAS (D1D3G, #15102 S, Cell Signaling Technology, 1:500), rabbit-anti-STING (D2P2F, #13647 S, Cell Signaling Technology, 1:500), rabbit-anti-phospho-STING (Ser366) (D7C3S, #19781 S, Cell Signaling Technology, 1:500), rabbit-anti-IRF3 (ab76409, Abcam, 1:1000), rabbi-anti-phospho-IRF3 (ab76493, Abcam, 1:1000), rabbit-anti-GAPDH (BE0024, EASYBIO, 1:2000), mouse-anti-β-Tubulin (CW0098, CWBIO, 1:2000), rabbit-anti-SARS-CoV-2 spike protein (40589-T62, Sino Biological, 1:1000), rabbit-anti-SARS-CoV-2 nucleocapsid protein (NP) (40143-R019, Sino Biological, 1:1000), rabbit-anti-Sendai virus (PD029C1, MBL, 1:1000), rabbit-anti-ACE2 (10108-T60, Sino Biological, 1:1000), mouse-anti-MAVS (sc166583, Santa Cruz, 1:500), goat-anti-mouse IgG-HRP secondary antibody (115035003, Jackson ImmunoResearch), mouse-anti-Lamin A/C (MABT538, Sigma-Aldrich, 1:500), rabbit-anti-Lamin B1 (ab16048, Abcam, 1:500), goat-anti-rabbit IgG-HRP secondary antibody (111035003, Jackson ImmunoResearch). For STING dimer detection, reducing reagents were not added to the lysates, and samples were not heated before loading.

### Real-time PCR

Total RNA was extracted using RNAprep Pure Micro Kit (DP420, TIANGEN) and reverse-transcribed using the Quantscript RT Kit (KR103, TIANGEN). Real-time quantitative PCR was performed using SYBR green kit (RR820A, Takara Bio). The following primers were used for real-time PCR: *GAPDH*-F: 5′-GGCATGGACTGTGGTCATGAG-3′, *GAPDH*-R: 5′-TGCACCACCAACTGCTTAGC-3′, *IFNB*-F: 5′-ACGCCGCATTGACCATCTAT-3′, *IFNB*-R: 5′-TAGCCAGGAGGTTCTCAACA-3′, *IL8*-F: 5′-CCACCGGAAGGAACCATCT-3′, *IL8*-R: 5′-GGCCAGCTTGGAAGTCATGT-3′, *ISG15*-F: 5′- GAGAGGCAGCGAACTCATCT-3,

*ISG15*-R: 5′-CTTCAGCTCTGACACCGACA-3′, *CCL5*-F:5′- CCCAGCAGTCGTCTTTGTCA-3′, *CCL5*-R: 5′- TCCCGAACCCATTTCTTCTCT-3′. *IFIT1*-F: 5′-TACAGCAACCATGAGTACAA-3′, *IFIT1*-R: TCAGGTGTTTCACATAGGC.

### Absolute quantitative PCR for viral *N* mRNA

Calu-3 or HeLa-ACE2 cells were treated with indicated drugs, followed by SARS-CoV-2 infection. At 24 h (Calu-3) or 48 h (HeLa-ACE2) post infection, supernatants were collected and subjected to RNA extraction using Direct-zol RNA MiniPrep kit (Zymo research, CA, USA) according to the manufacturer’s instructions. Viral copy numbers were measured by RT-PCR using primers and probes targeting the SARS-CoV-2 *N* gene. The reference standard was serially diluted by tenfold from 1 × 10^9^ copies to 1 × 10^4^ copies. PCR amplification procedure was 50 °C, 15 min, 95 °C, 3 min; 95 °C, 15 s, 60 °C, 45 s, 50 cycles, and data were processed by Bio-Rad CFX Manager software. The primer sequence specific for SARS-CoV-2 *N* was available from J.W. upon request.

### IC_50_ and CC_50_ determination

Calu-3 or HeLa-ACE2 cells were seeded in 96-well plates one day before infection. For IC_50_ determination, Calu-3 cells were pre-treated with drugs at concentrations of 0.0078, 0.0156, 0.03125, 0.0625, 0.125, 0.25, 0.5, 1, 2, and 4 μg/ml for 24 h, and HeLa-ACE2 were treated with drugs at concentrations of 0.013, 0.041, 0.123, 0.370, 1.111, 3.333, 10, and 30 μg/ml for 1 h. Cells were then subjected to viability assay or infected with SARS-CoV-2 at an MOI of 0.2. After 24 h (Calu-3) or 48 h (HeLa-ACE2), supernatants were harvested for RNA extraction, followed by absolute quantification of viral *N* mRNA by quantitative PCR. The inhibition ratio was obtained by dividing the viral copy number in drug-treated samples by those in the vehicle control samples. For CC_50_ determination, cells were pre-treated with each drug at indicated concentrations and time points. Cell viability was evaluated using a CCK8 kit (Yeasen, Beijing, China) according to the manufacturer’s instructions. The IC_50_ and CC_50_ values were calculated using Prism (Graphpad Software Inc.).

### Immunofluorescence

Cells were washed with PBS buffer and fixed with 4% paraformaldehyde (PFA) in PBS for 20 min at room temperature. Cells then were permeabilized with 0.5% Triton X-100 and blocked in a blocking buffer comprising 5% bovine serum albumin (BSA). Cells were stained with primary antibodies, followed by staining with second antibodies, including Alexa Fluor 488 AffiniPure Goat Anti-Rabbit IgG (H + L) (111545003, Jackson ImmunoResearch), Goat anti-Mouse IgG (H + L) Highly Cross-Adsorbed Secondary Antibody (A16080, Thermo Fisher), and Alexa Fluor Plus 647 (A32728, Thermo Fisher). The primary antibodies used in this research were: mouse-anti-SARS-CoV-2 Nucleoprotein (40143-MM08, Sino Biological), rabbit-anti-cGAS (D1D3G, #15102 S, Cell Signaling Technology), rabbit-anti-STING (D2P2F, #13647 S, Cell Signaling Technology), rabbit-anti-phospho-STING (Ser366) (D7C3S, #19781 S, Cell Signaling Technology), rabbit-anti-IRF3 (D6I4C, #11904 S, Cell Signaling Technology), mouse-anti-Calcineurin (C0581, Sigma-Aldrich), rabbit-anti-γH2AX (20E3, #9718 S, Cell Signaling Technology), mouse-anti-Flag (F3165, Sigma-Aldrich), mouse-anti-Lamin A/C (MABT538, Sigma-Aldrich), rabbit-anti-Lamin B1 (ab16048, Abcam), mouse-anti-HA (CW0092, CWBIO). Fluorescence images were obtained and analyzed using Leica Microsystems (LAS X) or Dragonfly Spin-disk system (ANDOR) on a Leica DMi8 microscope. Three-dimensional images were obtained from z-stack images collected at 0.2 μm intervals and processed by ImageJ. For live-cell imaging, HeLa-ACE2 cells were transfected with cGAS-GFP and were then co-cultured with HEK293T cells transfected with cGAS-GFP and spike. After 1 h, cells were stained with DRAQ5 (1:250) and subjected to live-cell imaging. Images were recorded every 1 min for 3.5 h and analyzed by the Fusion 2.0 software (ANDOR) and ImageJ.

### Quantification of 2′3′-cGAMP

SARS-CoV-2-infected cells were lysed in mPER lysis buffer (78501, Thermo Fisher) and heated to inactivate the virus. For 2′3′-cGAMP measurement, an ELISA kit based on the competition between 2′3′-cGAMP and a 2′3′-cGAMP-horseradish peroxidase conjugate (501700, Cayman Chemical) was used according to the manufacturer instructions.

### Animal experiments

The animal experiments were performed according to the protocols approved by the Institute of Animal Use and Care Committee of the Institute of Laboratory Animal Science, Peking Union Medical College (license no. BYS20016). Eight-week-old female hACE2 transgenic mice (GemPharmatech Co., Ltd) housed in the animal BSL3 (ABSL3) laboratory were anesthetized with tribromoethanol and inoculated intranasally with 10^5^ TCID_50_ of the SARS-CoV-2 virus in a volume of 40 μl diluted in phosphate-buffered saline (PBS). For histology and immunohistochemistry analysis, mouse lungs were removed on day 3, fixed with 4% formaldehyde, and embedded in paraffin. Lung tissue sections were produced, followed by staining with haematoxylin and eosin (H&E). Mouse-anti-SARS-CoV-2 nucleocapsid antibody (40143-MM08, Sino Biological), rabbit-anti-phospho-STING (Ser365) (D8F4W, #72971, Cell Signaling Technology) were used for immunohistochemical assays after antigen retrieval of lung tissue sections. Images of lung sections were obtained with Nikon LV2000 imaging system.

### Statistics

Statistical analysis was performed using Prism (Graphpad Software Inc.). The Student’s *t*-test was used for two-group comparisons. The values *P < 0.05, **P < 0.01, ***P < 0.001, and ****P < 0.001 were considered significant. n.s. stands for not significant.

## Supplementary information

Supplemental Movie 2

Supplementary Figures

Supplemental Movie 1

## Data Availability

On reasonable request, the corresponding author will provide all data supporting the findings of this study.
