# Supplementary material for: Sensing of cytoplasmic chromatin by cGAS activates innate immune response in SARS-CoV-2 infection
Source: Signal Transduct Target Ther. 2021 Nov 3;6:382. doi: 10.1038/s41392-021-00800-3 (PMC8564796; doi:10.1038/s41392-021-00800-3)
Supplement: Supplementary file 2 — Supplementary Figures [file 41392_2021_800_MOESM2_ESM.docx]

Supplementary Materials for

**Sensing of cytoplasmic chromatin by cGAS activates innate immune response in SARS-CoV-2 infection**

Zhuo Zhou, Xinyi Zhang, Xiaobo Lei, Xia Xiao, Tao Jiao, Ruiyi Ma, Xiaojing Dong, Qi Jiang, Wenjing Wang, Yujin Shi, Tian Zheng, Jian Rao, Zichun Xiang, Lili Ren, Tao Deng, Zhengfan Jiang, Zhixun Dou, Wensheng Wei, Jianwei Wang

Correspondence to: wangjw28@163.com, wswei@pku.edu.cn, zhouzhuo@gmail.com

**This PDF file includes:**

Figures S1 to S6


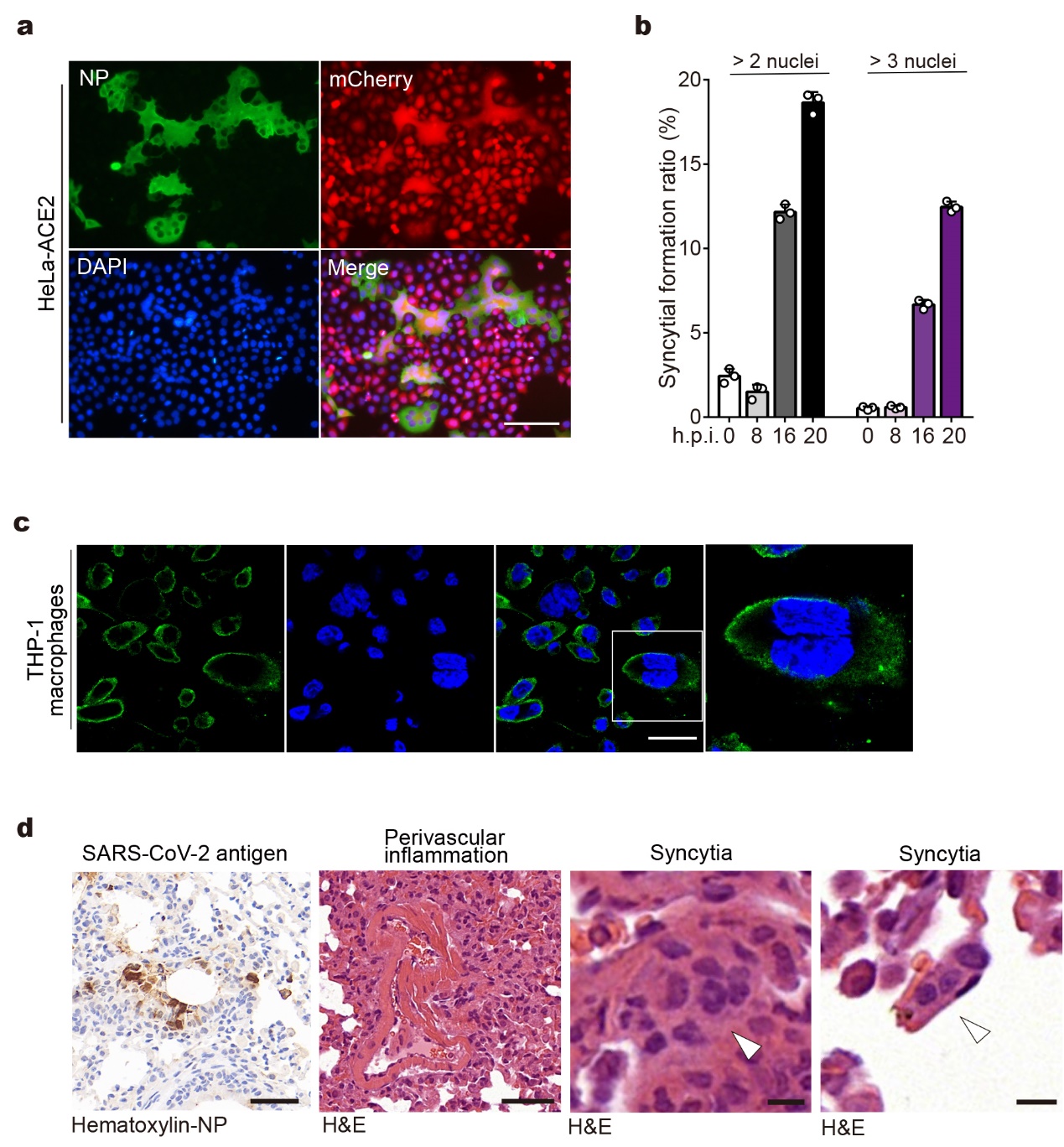


**Figure S1. SARS-CoV-2-induced syncytia formation**

**a** Representative immunofluorescence images of SARS-CoV-2-induced syncytia. HeLa-ACE2 cells stably expressing mCherry (HeLa-ACE2-mCherry) were infected with SARS-CoV-2 at an MOI of 0.5. After 18 h, cells were fixed and stained with DAPI (blue) and anti-NP antibody (green). Scale bar, 250 μm. **b** Syncytia formation ratio at different time points post SARS-CoV-2 infection. HeLa-ACE2-mCherry cells were infected with SARS-CoV-2 at an MOI of 0.5 for indicated times. Syncytia formation ratios were calculated by dividing mCherry positive cells containing more than two or three nuclei by total mCherry positive cells using a high content imaging instrument. **c** Representative immunofluorescence images of SARS-CoV-2-induced syncytia in THP-1 macrophage cells. Macrophage-like cells differentiated from THP-1 cells using phorbol-12-myristate-13-acetate (PMA) were infected with SARS-CoV-2 at an MOI of 0.5. After 18 h, cells were fixed and stained with DAPI (blue) and anti-NP antibody (green). Scale bar, 40 μm. **d** Syncytia formation in mice infected with SARS-CoV-2. K18-hACE2 transgenic mice were infected with 10^5^ TCID_50_ of the SARS-CoV-2 for 3 days. Mouse lungs were harvested and subjected to histology or immunohistochemistry analysis as indicated. Scale bars in images showing NP staining and perivascular inflammation, 50 μm. Scale bars in images showing syncytia, 10 μm.


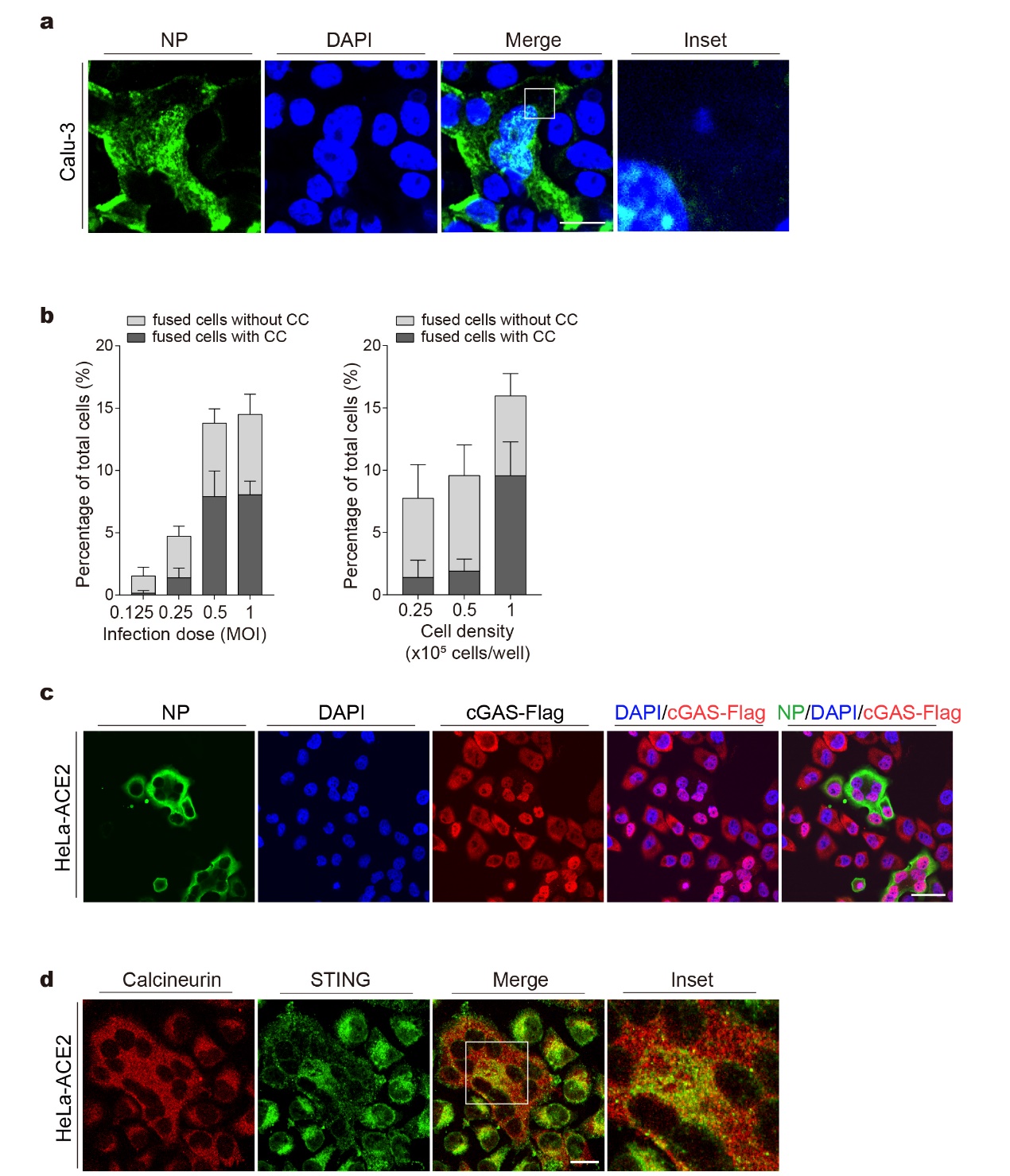
c

**Figure S2. SARS-CoV-2-induced cytoplasmic chromatin and cGAS/STING translocation**

**a** Representative images of cells stained for NP and DNA. Calu-3 cells were infected with SARS-CoV-2 at an MOI of 0.5 for 18 h, followed by staining with DAPI (blue) and anti-NP antibody (green). Scale bar, 20 μm. **b** Quantification of cells for parameters as indicated. Left panel, HeLa-ACE2 cells at ~90% confluency were infected with SARS-CoV-2 at indicated MOIs. After 18 h, cells were fixed and stained with DAPI and anti-NP antibody, followed by fluorescence microscopy analysis. Right panel, HeLa-ACE2 cells were seeded in 24-well plate at indicated density. Cells were then treated as described in the left panel. CC, cytosolic chromatin. Mean ± s.d., n = 3 independent experiments. **c** Representative images of cells stained with anti-NP antibody (green), DAPI (blue), and anti-Flag antibody (red). cGAS-null HeLa-ACE2 cells reconstituted with cGAS-Flag were treated as described in (**a**), followed by staining with antibodies as indicated. Scale bar, 40 μm. **d** Representative images of cells stained for ER and STING. HeLa-ACE2 cells were treated as described in (**a**) and were stained with anti-Calcineurin (red) antibody and anti-STING (green) antibody as indicated. Scale bar, 20 μm.


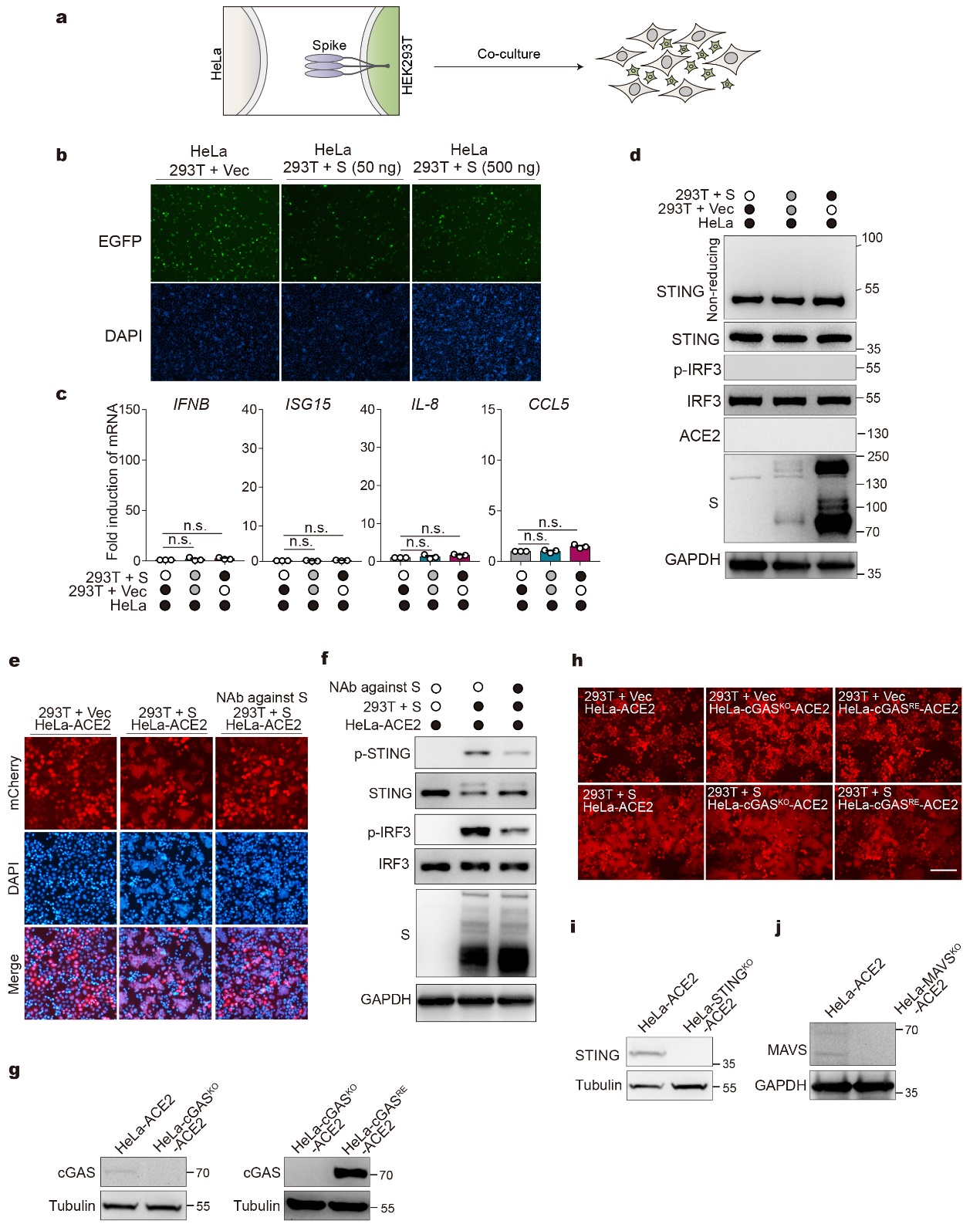


**Figure S3. Activation of innate immune response by cell co-culture**

**a** Scheme of co-culture experiment. **b** Representative fluorescence images of co-culture experiment. HEK293T cells were transfected with vector control (Vec) or increasing amounts of plasmids expressing spike (S), along with plasmids expressing EGFP. After 24 h, cells were detached and mixed with wildtype HeLa for 8 h. Cells were then subjected to fluorescence microscopy analysis. Scale bar, 250 μm. **c** Cytokine genes/ISGs expression in co-cultured cells. Cells were co-cultured as indicated in (**b**). RNA extracted from the cells was evaluated by quantitative PCR. The data are expressed as fold change of the *IFNB*, *ISG15*, *IL8*, and *CCL5* mRNA levels relative to the *GAPDH* control. Mean ± s.d., n = 3. n.s., not significant. two-tailed Student’s *t*-test. **d** Western blot analysis of cells from co-culture experiment as described in (**b**) using indicated antibodies. **e** Inhibition of cell fusion by an anti-spike antibody. HEK293T cells were transfected with vector control (Vec) or plasmids expressing S. After 24 h, cells transfected with S were left untreated or treated with a neutralizing antibody against spike at a concentration of 50 μg/ml. After 12 h, cells were detached and mixed with HeLa-ACE2 cells for 5 h. Cells were then subjected to fluorescence microscopy analysis as indicated. Scale bar, 200 μm. **f** Western blot analysis of cells from co-culture experiment as described in (**e**) using indicated antibodies. **g** cGAS expression level in Hela-ACE2, HeLa-cGAS^KO^-ACE2, and HeLa-cGAS^RE^-ACE2 cells. **h** HEK293T cells were transfected with vector control (Vec) or plasmids expressing S. After 24 h, cells were detached and mixed with HeLa-ACE2-mCherry cells (wildtype, cGAS^KO^, or cGAS^RE^) for 8 h. Cells were then subjected to fluorescence microscopy analysis. Scale bar, 250 μm. **i** STING expression level in HeLa-ACE2 and Hela-STING^KO^-ACE2 cells. **j** MAVS expression level in HeLa-ACE2 and HeLa-MAVS^KO^-ACE2 cells.


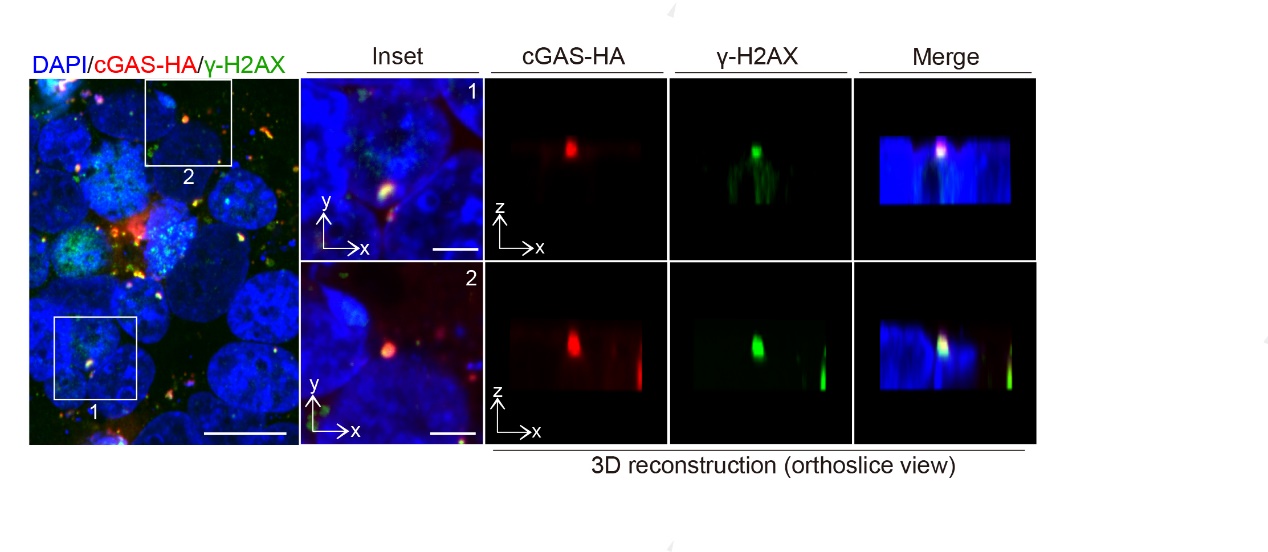


**Figure S4. cGAS is colocalized with γH2AX in syncytial cells**

Co-culture experiments were performed as described in Fig. 4e. Cells were stained with DAPI (blue), anti-HA (red) antibody, and anti-γH2AX (green) antibody. Three-dimensional reconstructed images were displayed as orthoslice views. Scale bar, 20 μm or (inset) 5 μm.


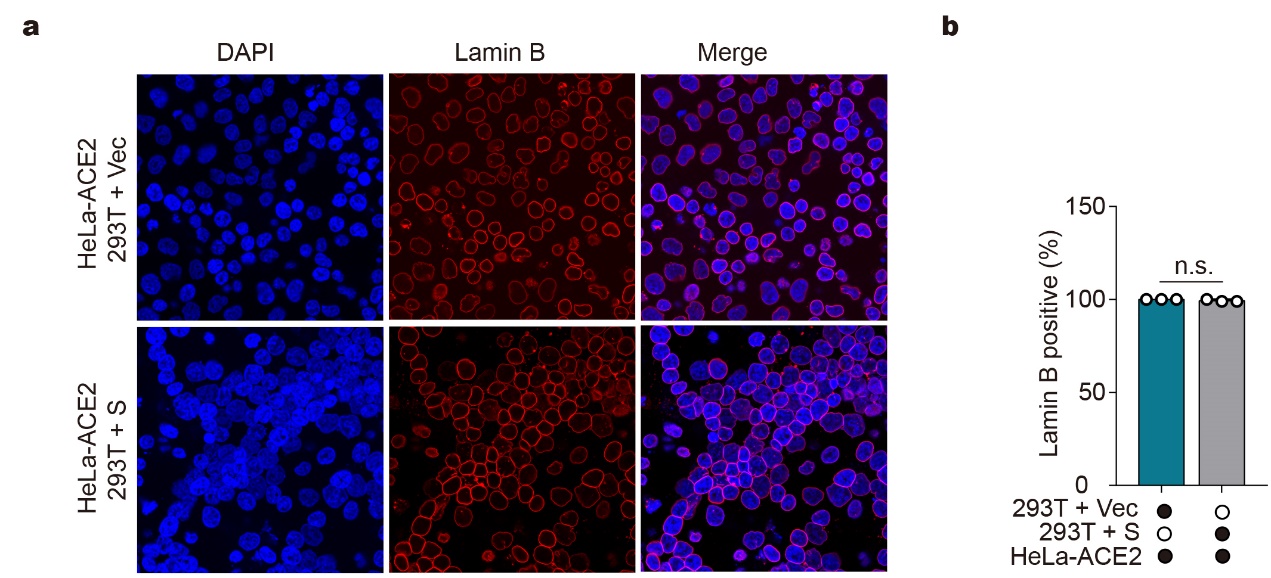


**Figure S5. Lamin B expression in co-cultured cells.**

**a** Representative fluorescence images of co-culture experiment. HEK293T cells were transfected with vector control (Vec) or plasmids expressing S for 24 h. Cells were then detached and mixed with HeLa-ACE2. After 4.5 h, cells were stained with DAPI (blue) and anti-Lamin B antibody as indicated. Scale bar, 20 μm. **b** Quantification of Lamin B positive cells. Mean ± s.d., n = 3 independent experiments. n.s., not significant, two-tailed Student’s t-test.


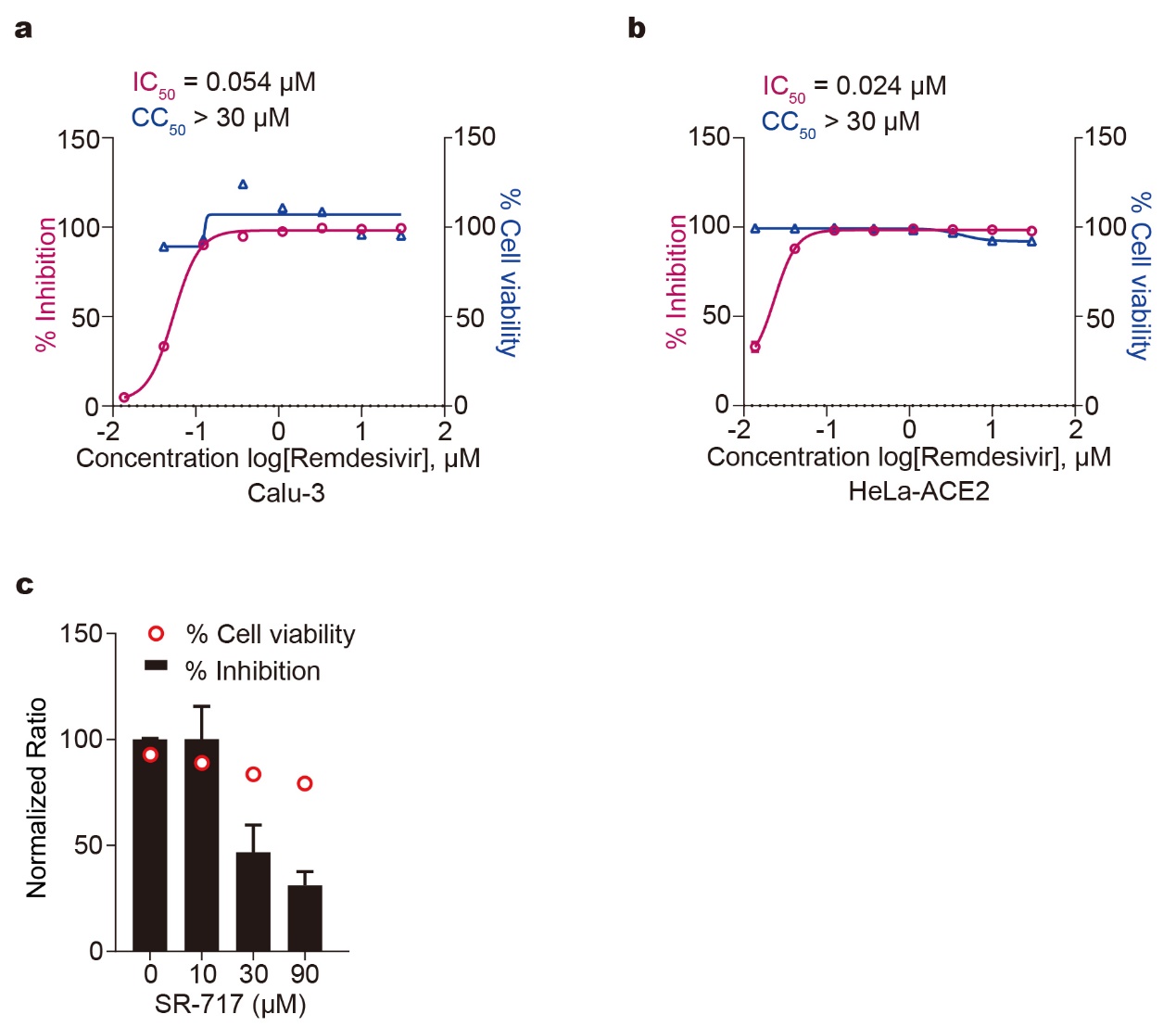


**Figure S6. The antiviral effect of Remdesivir and SR-717 on SARS-CoV-2**

**a**, **b** Cells were treated with serially diluted Remdesivir for 24 h (Calu-3) or 1 h (HeLa-ACE2). Cells were then subjected to viability assay or infected with SARS-CoV-2 at an MOI of 0.2. After 24 h (Calu-3) (**a**) or 48 h (HeLa-ACE2) (**b**), supernatants were harvested for RNA extraction, followed by absolute quantification of viral *N* mRNA by PCR. The IC_50_ (The half-maximal inhibitory concentration) and CC_50_ (The half-maximal cytotoxic concentration) values were calculated using Prism software. **c** Calu-3 were treated with SR-717 at indicated concentrations for 24 h. Cells were then subjected to viability assay or infected with SARS-CoV-2 at an MOI of 0.2. After 24 h, supernatants were harvested for RNA extraction, followed by absolute quantification of viral *N* mRNA by PCR.
